# Distal Arthrogryposis and Lethal Congenital Contracture Syndrome – An Overview

**DOI:** 10.3389/fphys.2020.00689

**Published:** 2020-06-25

**Authors:** Darshini Desai, Danielle Stiene, Taejeong Song, Sakthivel Sadayappan

**Affiliations:** Division of Cardiovascular Health and Disease, Department of Internal Medicine, Heart, Lung and Vascular Institute, College of Medicine, University of Cincinnati, Cincinnati, OH, United States

**Keywords:** distal arthrogryposis, LCCS4, striated muscle, *MYBPC1*, *MYBPC2*, titin

## Abstract

Distal arthrogryposis (DA) is a skeletal muscle disorder which can be classified under a broader term as Arthrogryposis multiplex contractures. DA is characterized by the presence of joint contractures at various parts of the body, particularly in distal extremities. It is identified as an autosomal dominant and a rare X-linked recessive disorder associated with increased connective tissue formation around joints in such way that immobilizes muscle movement causing deformities. DA is again classified into various types since it manifests as a range of conditions representing different etiologies. Myopathy is one of the most commonly listed etiologies of DA. The mutations in sarcomeric protein-encoding genes lead to decreased sarcomere integrity, which is often associated with this disorder. Also, skeletal disorders are often associated with cardiac disorders. Some studies mention the presence of cardiomyopathy in patients with skeletal dysfunction. Therefore, it is hypothesized that the congenitally mutated protein that causes DA can also lead to cardiomyopathy. In this review, we will summarize the different forms of DA and their clinical features, along with gene mutations responsible for causing DA in its different forms. We will also examine reports that list mutations also known to cause heart disorders in the presence of DA.

## Introduction

Arthrogryposis is a common disorder characterized by the development of non-progressive contractures affecting the muscles congenitally. It is coined as arthrogryposis multiplex contractures – multiplex because it affects multiple parts of the body (Sucuoglu et al., 2015). Multiple congenital joint contractures are classified into amyoplasia, distal arthrogryposis (DA), and syndromic. Distal Arthrogryposis, as the name suggests, affects the distal parts of the limbs, and it can occur in the absence of any primary neurological disorder, or, indeed, any type of muscular disorder (Bamshad et al., 2009). Most symptoms involve contractures affecting two or more areas of the body with least involvement of the proximal joints. It is highly lucid, meaning that symptoms often vary among individuals of the same family presenting with the same disorder. DA is associated with syndromes like Freeman-Sheldon syndrome (FSS), Gordon syndrome, Sheldon-Hall syndrome, multiple pterygium syndrome and trismus-pseudocamptodactyly syndrome. As the syndrome is associated with a multiplicity of other conditions, causation varies from type to type. However, the most common etiology is reduced fetal movement which leads to the formation of congenital contractures. These are very severe since decreased fetal movement causes extra connective tissue formation around joints (Gordon, 1998). Previous reports suggest that fetus movement is compromised by the lack of space in the uterus, which then causes impaired vascular supply to the fetus. Other major etiologies associated with DA include neurological abnormalities, muscle abnormalities, and abnormalities in the formation of connective tissues. Sometimes maternal diseases can also contribute to the development of this syndrome (Hall, 1997). The overall objectives of this review article are to provide an extensive overview of DA and its clinical, genetic and molecular aspects, and determine opportunities to expand research studies to cure DAs.

## Clinical Features of Distal Arthrogryposis

To date, more than ten different types of Distal Arthrogryposis have been identified. They are classified as Distal Arthrogryposis Type 1 (DA1), Distal Arthrogryposis Type 2A (DA2A) and 2B (DA2B), and Distal Arthrogryposis Types 3–10 (DA3–10) (Bamshad et al., 2009). Each type is further classified according to its clinical features and pathology.

**FIGURE 1:**
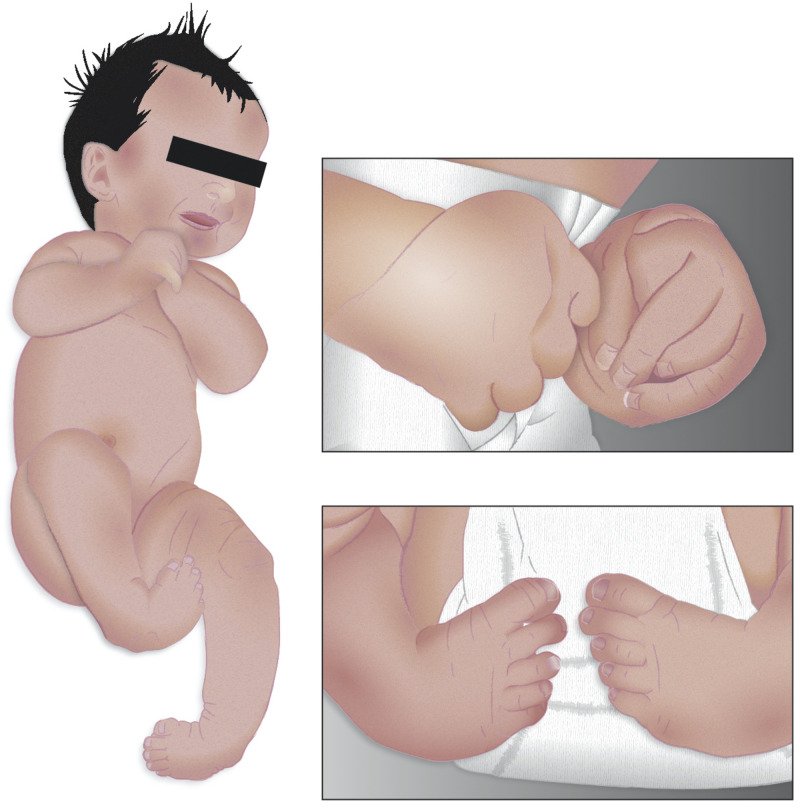
Morphological features of distal arthrogryposis. A child born with distal arthrogryposis, showing characteristic camptodactyly, such as one or more fingers permanently bent. The characteristic clubfoot is also seen, as well as the abnormal finger positions of the hand (Modified from Chen, 2015).

**Distal Arthrogryposis Type 1** sometimes overlaps with a disorder called FSS owing to the similarity of respective clinical features (Gurnett et al., 2010; Alvarado et al., 2011; Beck et al., 2013; Wang et al., 2016; Jin et al., 2017; Poling et al., 2017). The prevalence of DA is estimated to be ∼1 in every 3,000 people worldwide (Hall et al., 1982; Bamshad et al., 1996; Beals, 2005), and it is more common than other types. It is characterized by the presence of camptodactyly, meaning that patients have bent fingers and toes (Figure 1). Patients also suffer from a type of hand deformity such that all fingers are angled outward toward the fifth finger, termed as ulnar deviation, and they have fingers that overlap. Intelligence is not said to be compromised in patients with this syndrome, nor do neurological reports show any abnormalities (Bamshad et al., 1996). Other clinical features include the presence of clubfoot, which is an inward- and downward-turning foot (Figure 1). These patients have a triangular face with prominent nasolabial folds, downward-slanting palpebral fissures, and other calcaneovalgus deformities (Krakowiak et al., 1997).

**Distal Arthrogryposis Type 2** is further classified into two groups termed as DA2A (FSS) and DA2B (Sheldon-Hall syndrome). DA2B is an intermediary phenotype between DA1 and FSS. DA2B is also referred to as a variant of FSS (Krakowiak et al., 1998). Clinical features are likely similar to those of DA1, including ulnar deviation, overriding fingers at birth and camptodactyly. Major clinical features for this type are related to lower limbs and consist of talipes equinovarus (clubfoot), calcaneovalgus deformities, vertical talus (flatfoot), and metatarsus varus (Guell et al., 2015; Li et al., 2015; Staff, 2015). Minor features include triangular face, descending-slanting palpebral fissures, joined ear lobules, small mouth, small mandible, arched palate, webbed neck, and shorten height (Krakowiak et al., 1997).

**Distal arthrogryposis type 3** (Gordon syndrome) is a rare and inherited disorder that affects movement in the joints of the upper and lower limbs (Hall et al., 1982). Patients are born with stiff joints that function improperly and are difficult to move. They also suffer from camptodactyly and clubfoot. However, this type of DA is distinct from others by the presence of shorten height and cleft palate (Bamshad et al., 2009). The range and severity of these features can vary from patient to patient. Usually, the patient’s intelligence is unaffected and mostly normal (Poling et al., 2017). Other clinical features include rigid fingers, bilateral congenital clubfoot, constrained horizontal and vertical eye movements, inflexible back, stiff walk, anteverted slouched shoulders, and pectus excavatum (Schrander-Stumpel et al., 1993).

**Distal arthrogryposis 4** (scoliosis) **and 6** (sensorineural hearing loss) are newer and rarer types. They are scarcely studied; therefore, only a very few reports are available. However, they do have unique clinical features, making them distinct from other types of DA. Specifically, DA4 is characterized by the presence of curvature of the spine, which is a critical feature (Bamshad et al., 2009). Scoliosis is often described as a sideway curvature of the spine, which is inherited in an autosomal dominant manner. Other clinical features include camptodactyly and torticollis, i.e., twisted neck, where patients have their neck tilted to one side (Baraitser, 1982). DA6 is distinguished by the presence of hand anomaly and sensorineural deafness. These clinical features range from moderate to severe, and the disorder is often inherited from males.

**Distal arthrogryposis type 5** (ophthalmoplegia) is another type of DA characterized by the presence of ptosis/oculomotor dysfunction. This is not always true as affected individuals have also been known to show some non-ocular features. They also show contractures of the distal joints, limited ocular movements, peculiar facial highlights with deep−set eyes, and shortening of the first and fifth toes. Sometimes patients also have restrictive lung failure which is recognized as a syndromic component of DA5 in adults (Okubo et al., 2015).

**Distal arthrogryposis type 7** (trismus-pseudocamptodactyly) involves in failure to open the mouth fully (trismus) which complicates dental alignment and care, feeding at post-natal stage, and intubation for anesthesia. It also shows the presence of pseudocamptodactyly in which the wrist causes flexion contracture of distal and proximal interphalangeal joints causing occupational and social disability (Veugelers et al., 2004; Minzer-Conzetti et al., 2008).

**Distal arthrogryposis type 8** (autosomal dominant multiple pterygium syndrome, DA8) clinical features include contractures of proximal and distal joints, pterygia involving the neck, axillae, elbows, and knees. Researchers performed exome sequencing in three families with similar clinical features like DA8 and identified a heterozygous mutation in the *MYH3* gene (Chong et al., 2015). Mutations in these families were highly conserved residues and hence predicted to play a major role in developing the DA8-like phenotype. Many other studies also confirmed that mutations in the *MYH3* contribute to the development of DA8. Altogether, DA8 is termed as an autosomal dominant type of DA attributed to mutation in the *MYH3* gene (Zieba et al., 2017; Cameron-Christie et al., 2018; Scala et al., 2018).

**Distal arthrogryposis type 9** (congenital contractural arachnodactyly, DA9) is also called congenital contractural arachnodactyly (CCA) and Beals syndrome named after the researcher who first studied it (Beals and Hecht, 1971). It is noted by several researchers that CCA is caused by heterozygous mutation in the fibrillin-2 gene (FBN2) (Wang et al., 1996; Park et al., 1998). CCA is an uncommon autosomal dominant disorder described by contractures, abnormally long fingers, scoliosis, and scrunched ears (Hecht and Beals, 1972). The clinical features of DA9 overlap with Marfan syndrome which is attributed to mutations in fibrillin-1, *FBN1*. In a comparison between Marfan syndrome and DA9, one researcher reported that the abnormally shaped auricular helices were the trademark of CCA and, hence, absent in individuals with Marfan syndrome (Godfrey et al., 1995). Both syndromes are said to have clinically different structural and functional characteristics. For instance, one study suggests that structural defects, such as complete closure of the lumen of the duodenum, esophageal atresia, and intestinal malrotation, are evidently seen in CCA. In contrast, Marfan syndrome has more functional defects like valvular insufficiency and aortic root dilatation (Wang et al., 1996). This is mainly attributed to mutations in the proteins found in these syndromes. For example, Fibrillin-2 mediates the assembly of elastic fibers at prenatal stages, whereas fibrillin-1 provides the stiffness to the microfibrils (Zhang et al., 1994, 1995).

**Distal arthrogryposis type 10** (congenital plantar syndrome, DA10) is a rare genetic disease characterized by plantar flexion contractures, presenting with toe-walking in infancy and variably associated with milder contractures of the hip, elbow, wrist, and finger joints (Levine, 1973). No ocular or neurological abnormalities are associated with DA10 (Hall et al., 1967), similar to other DAs, but this type is still poorly studied. However, in a study conducted on a Utah family of five generations, multiple individuals showed plantar flexion contractures in an autosomal dominant, and the author termed it as DA10. Onset was typically in early childhood and manifested as toe walking. Contractures of joints often involved the elbows, but nerve and bone morphology appeared to be normal. However, family members with wrist contractures could still function normally (Stevenson et al., 2006).

## Current Treatments for Distal Arthrogryposis

The primary goal for the treatment of the patients with DA is to improve their quality and quantity of life toward independent living. This requires help in improving the motor function of any affected joints, strengthening any functional muscles, and correcting any fixed deformities that affect daily activities (Kowalczyk and Felus, 2016). A variety of treatment tools are used, the first being rehabilitation, which includes physiotherapy, manipulation of contractures, and occupational therapy. More targeted and individually designed treatments like orchestrated orthotic management is done to prevent any repeated deformities. However, the most preferred option for treatment is surgical correction of musculoskeletal deformities. For upper limbs like the shoulder joint, a subcapital derotation osteotomy of the humerus is performed, usually in severe internal rotation contractures (Zlotolow and Kozin, 2012). The presence of extended contracture of the elbow joint makes routine activities very difficult. The treatment for such patients includes capsulotomy which helps improve passive elbow flexion, active flexion by triceps, or both (Axt et al., 1997). Patients with contractures related to knee joints are very common and those with severe knee dislocations usually require surgical treatment that has to be done in the early stages. Surgical procedures to correct such deformities vary, depending on the severity of the contractures and age of the patients. A few examples are as follows: soft-tissue release, femoral shortening-extension surgery, correction with Ilizarov, which is an external fixation to lengthen or reshape limb bones, and femoral anterior epiphysiodesis, which involves surgical intervention to stop bone growth around the knee. Many clinicians prefer surgical options like percutaneous quadriceps tenotomy, in which the tendon is cut to correct the deformity, open quadricepsplasty to improve knee flexion, and femoral shortening osteotomy (Lampasi et al., 2012). The Ponseti method of manipulation has been successfully performed on pediatric patients to correct clubfoot, and this procedure is followed by Achilles tenotomy. More major surgeries can then be avoided in these patients, and the above method has also proven to be a good initial treatment in children with DA (Kowalczyk and Felus, 2016).

## Gene Mutations Linked to Distal Arthrogryposis

**FIGURE 2:**
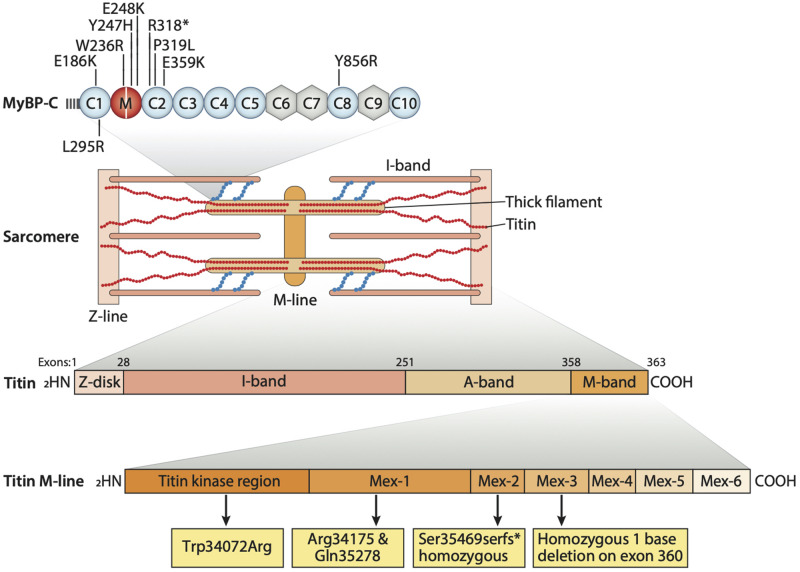
Sarcomere structure containing sarcomeric proteins responsible for proper function. Various regions of titin consisting of Z-disk, I-band, A-band, and M-band. M-line region of titin consists of the titin kinase region (Tk region). In the top panel, known mutations in *MYBPC1* gene are listed. Patients with TK mutation (Trp34072Arg) presented dilated cardiomyopathy and DA phenotype. Mutations in Mex-1 Arg34175 and Gln35278* show DCM and joint contractures. Mutations in Mex-2 Ser35469serfs* show DCM and moderate to severe joint contractures. Homozygous 1 base deletion on exon 360 also presents with skeletal muscle disorder and fetal cardiomyopathy.

Distal arthrogryposis type 1 is said to involve at least two genes, namely *TPM2 (β-tropomyosin)* and *MYBPC1 (Slow skeletal myosin binding protein-C)*. These genes are expressed in muscle cells, and their interaction with sarcomeric proteins helps in regulating muscle contraction (Figure 2). We still do not know how these two gene mutations can lead to the formation of joint contractures characteristic of DA type 1. However, some researchers suggest that the contractures result from a lack of movement in the fetus which, in turn, leads to improper muscle tissue formation. Nevertheless, researchers are still searching for genetic changes that can cause this condition (Sung et al., 2003). Another mutation linked to DA2B involves the *TNNI2* gene which plays a role in affecting the C’-terminal domain of troponin I (TnI) and alters troponin-tropomyosin (Tc-Tm) complex (Sung et al., 2003). To assess the effects of human DA mutations, *MYBPC1* mRNAs, resembling W236R and Y856H mutations, were administrated into zebrafish embryos, after which mild bent body curvatures and decreased motor activity were observed. Another skeletal myosin binding protein-C paralog, *MYBPC2* (fast skeletal myosin binding protein-C), is also involved in developing a severe form of DA (Bayram et al., 2016). Gene set enrichment analysis (GSEA) in over 400 different DA cases showed the association of *MYBPC2* mutation with DA (Hall and Kiefer, 2016). In a patient with unclassified DA, compound heterozygous mutations of *MYBPC2* were found with homozygous GPR126 mutation (19C > T, Arg7X) known to cause postnatal death (Langenhan et al., 2013). *MYBPC2* mutations (T236I and S255T) in patients are highly conserved in vertebrates and localized in the protein’s N-terminal region, the binding site of myosin S2 which is crucial for the regulation of thick and thin filaments (Bayram et al., 2016). Recently, two new heterozygous *MYBPC2* variants (V307A and A1065V) were also reported in a patient with DA (Pehlivan et al., 2019). In the zebrafish model, *MYBPC2* protein knockdown by morpholino antisense nucleotides (>50%) reduced sarcomere length and muscle strength with significantly increasing expressions of atrophic gene (Li et al., 2016). According to the studies, these sarcomeric gene mutations might be causing or exacerbating DA by either altering sarcomere integrity or by changing the muscle contractility through their interaction with other sarcomeric proteins (Ha et al., 2013). DA3 is reported to be caused by mutations in the piezo-type mechanosensitive ion channel component 2 (*PIEZO2)* gene, which are also likely to be involved in causing DA type 5 and Marden-Walker syndrome. This gene is known to encode a mechanosensitive ion channel, negatively affecting joints, ocular muscles, lung function, and bone development (McMillin et al., 2014). Titin is another sarcomeric protein and one considered to be the largest protein known in the body (Figure 2). It plays an integral role in changing the integrity of sarcomeric structure and its stability. It spans the half-sarcomere from Z-disk to M-band. It is also known to take part in the development of cardiac and skeletal muscle, and it is encoded by the 363-exon titin gene called *TTN*. Many studies have reported on the mutations in titin found in patients with skeletal disorders. Various mutations in the M line region of *TTN* can be linked to congenital myopathy and cardiac disorders (Figure 2). Also, mutations affecting the serine/threonine kinase region of titin, a very conserved and important region for gene expression and cell signaling in heart development, have been reported in patients suffering from DA and cardiac hypertrophy (Carmignac et al., 2007; Chauveau et al., 2014). Overall, the genetic and molecular aspects of DA remain to be studied systematically.

## Da and Heart Disease

As mentioned above, the mutations in titin are reported to play a role in causing skeletal and cardiac disorders. A study performed in 2007 discovered two homozygous deletions in M-line *TTN* gene (Mex1 and Mex3 exons) resulting in truncation of the protein’s C-terminal. The pathophysiological effects of these mutations helped gain insight into the role and importance of M line stability in titin. Titin is known to take part in organizing the sarcomeric proteins during myofibrillogenesis. The resultant truncated form of titin, owing to mutations, gets incorporated into the sarcomere and can cause disease. This clue led the authors to speculate that any critical disruptions in the C-terminal of titin could, theoretically, lead to decreased M line stability and sarcomere disarrangements, thus increasing the mechanical load on heart muscles. Such constant mechanical stress on heart muscle could then be considered as a probable mechanism contributing to the phenotype related to myopathy and heart disease (Carmignac et al., 2007).

**FIGURE 3:**
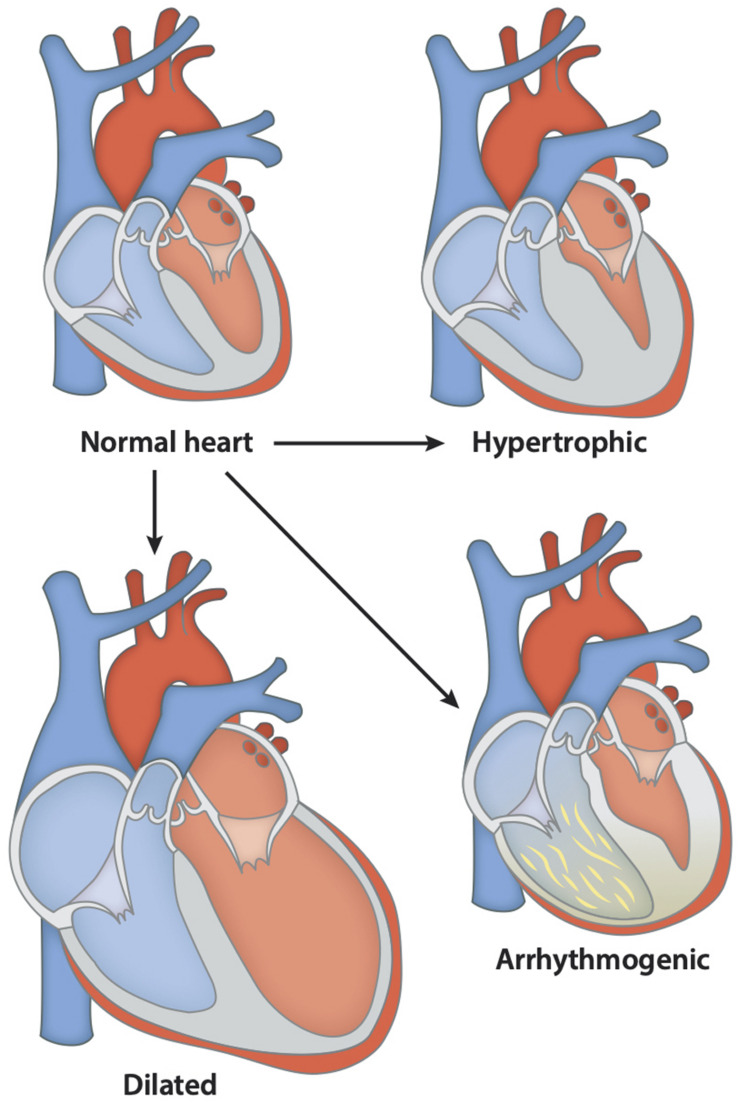
Various forms of cardiomyopathies associated with the development of DAs. The different forms of cardiomyopathy caused by different mutations shown in Table 1. These lead to morphological changes in the heart and dysfunction. In the above figure, mutations can lead to hypertrophic heart where the ventricular wall thickens, and the ventricular chamber gets smaller, leading to cardiac dysfunction. In contrast, the dilated heart shows thinner ventricular walls and bigger ventricular chamber, leading to reduced cardiac function. Arrhythmogenic heart is characterized by the presence of fat or fibrous tissue causing abnormal heart rhythms and, in severe cases, sudden cardiac arrest.

Another study done in 2014 further explored these *TTN* mutations believed to play a role in the etiology of skeletal disorders and cardiomyopathies (Figure 3). The study consisted of 31 patients who showed the presence of congenital core myopathy and primary heart disease. Subjects with genes typically related to myopathy and cardiac disease, such as *FHL1, LMNA, LAMP2, MYBPC3, ACTA1, MYL2, MYL3, PRKAG2, MYH7, TNNT2, TPM1, DES*, and *RYR1*, were excluded from the study. Sanger sequencing of the *TTN* M-line-encoding exons from Mex1 to Mex6 was performed (Figure 2). Results showed two patients homozygous for a two base-pair deletion in Mex2 (pSer35469Serfs^∗^11) presumed to produce a truncated titin protein at its C-terminal. Another patient also showed two heterozygous nonsense mutations in Mex1, p.Arg34175^∗^ and p.Gln35278. Using immunohistochemistry, a truncated titin was shown to be incorporated in a normal-looking sarcomere. Interestingly, two other patients were found to have mutations in Mex1, p.Asn34020Thrfs^∗^9 and p.Trp34072Arg, constituting a paternally inherited mutation critically affecting titin’s TK domain by causing deletions of its many C-terminal amino acids (Figure 2). The presence of a paternal mutation was suspected to be a recessive inheritance, causing a second mutation in these patients, and the authors concluded that this was responsible for the disease phenotype. As reproduced in Table 1, we can see how the authors link these mutations to different pathophysiologies seen in these patients. They also proposed a diagnostic screen to look for any mutations in six M-line-encoding *TTN* exons in patients who showed early-onset myopathies and cardiomyopathy (Chauveau et al., 2014).

**TABLE 1:** Genes and mutations associated with both skeletal and cardiac disorders.

**Gene**	**Mutations**	**Cardiac disorder**	**Skeletal muscle disorder**	**References**	**Organism**
*Mybpc1*	Humanized W236R and Y856H	Cardiac enlargement, low heart rate	Mild bent body curvatures and decreased motor activity	Ha et al., 2013	Zebrafish
*TTN*	pSer35469Serfs*11 homozygous	Dilated cardiomyopathy	Elbow and ankle joint contractures	Chauveau et al., 2014	Human
*TTN*	p.Arg34175* p.Gln35278	Dilated cardiomyopathy	Severe elbow and ankle joint contractures, scoliosis, neonatal hypotonia	Chauveau et al., 2014	Human
*TTN*	p.Asn34020Thrfs*9	Arrhythmia and dilated cardiomyopathy	Ankle joint contractures, scoliosis	Chauveau et al., 2014	Human
*TTN*	p.Trp34072Arg	Terminal heart failure and dilated cardiomyopathy	Distal arthrogryposis with knee, hip, digit and elbow contractures, neonatal hypotonia, dislocated hips	Chauveau et al., 2014	Human

*This table summarizes the different specific gene mutations and their cardiac disorder phenotypes. These mutations are also linked to skeletal muscle disorders, as these proteins are found in different muscle types throughout the body.*

Studies have suggested the involvement of *MYBPC1* in causing DA type1 and 2 (Li et al., 2015). *MYBPC1*, which is primarily found in slow-twitch skeletal muscles in humans, was also found to be present in zebrafish heart. This study on a zebrafish model of DA with human W236R and Y856H *MYBPC1* mutation showed cardiac edema, low pulse and cardiac hypertrophy (Ha et al., 2013). This study indicates the possible role of *MYBPC1* mutation in causing cardiac disorder, along with skeletal disorder.

## Lethal Congenital Contracture Syndrome and Its Features

Lethal congenital contracture syndrome (LCCS) is a rare severe and lethal class of arthrogryposis multiplex contracture syndrome. LCCS is an autosomal recessive syndrome, in comparison to Distal Arthrogryposis, which is autosomal dominant. Akinesia of the limbs and degeneration of motoneurons are main characteristics of LCCS. These clinical manifestations are accompanied by malformed joints and limbs. Eleven subtypes of LCCS have been known and described. LCCS1 is, as mentioned before, an autosomal recessive and neonatally lethal form of LCCS. It is characterized by the presence of congenital non-progressive joint contractures involving the upper and lower limbs and, sometimes, vertebral column. This leads to flexion or extension limitations, very evidently seen at birth (Makela-Bengs et al., 1998). Subtype 1 can be identified as early as week 13 of pregnancy *via* sonogram, as the fetus shows total akinesia with malformed limbs (Nousiainen et al., 2008). Other clinical symptoms of LCCS are incomplete lung development and fluid collection in the body. If born, neonates can die quickly from respiratory distress, other neurological deficits, and from akinesia. LCCS1 is caused by homozygous or compound mutation in GLE1 (GLE-like protein), which is required for export of mRNAs in eukaryotic cells (Nousiainen et al., 2008). LCCS2 is different from other subtypes owing to the presence of craniofacial/ocular deformities, lack of hydrops, multiple pterygia, distended urinary bladder, and other urinary abnormalities. Duration of pregnancy appears to be normal, but the prenatal diagnosis is possible as early as the 15th week of gestation (Landau et al., 2003). Researchers found a homozygous mutation in the ERBB3 (erb-b2 receptor tyrosine kinase 3) gene in affected members of a large cognate and in an isolated case (Narkis et al., 2004).

Lethal congenital contracture syndrome type 3 is also an autosomal recessive LCCS differing from LCCS2 by the lack of distended bladder. Affected individuals showed multiple joint contractures, and muscle atrophy. Individuals die within few minutes post-birth owing to respiratory failure. The phenotype can also be well distinguished from LCCS1 by the absence of body fluid accumulation, fractures, and facial anomalies. LCCS3 is caused by a homozygous missense mutation in the PIP5K1C gene, encoding a member of the type I phosphatidylinositol-4-phosphate 5-kinase family of enzymes. The mutation can affect a very conserved residue on the enzyme and abolishes its kinase activity (Narkis et al., 2004). LCCS4, specifically, is caused by a mutation in *MYBPC1*. This gene encodes *MYBPC1* found in slow and fast-twitch skeletal muscle. This is a nonsense mutation, causing truncation and non-functioning of the MYBPC1 protein. The mutation occurs in the C2 domain of MYBPC1 and causes malformation of the skeletal muscles. This homozygous mutation causes a similar, yet more severe, phenotype than the mutation that causes DA (Markus et al., 2012).

Lethal congenital contracture syndrome type 5 is also a lethal congenital neuromuscular syndrome known to be caused by homozygous mutations in the DNM2 gene, encoding for protein dynamin-2. A study reported that LCCS5 exhibited fetal movements, polyhydramnios, and decreased birth weight. However, at birth, all affected individuals showed severe hypotonia with respiratory insufficiency, lack of reflexes, retinal hemorrhages, joint contractures, and thin ribs and bones. Death associated with this syndrome is reported to occur at 5 days, 19 days, and 4 months after birth. Parents of these infants showed decreased reflexes on examination, and maternal skeletal muscle biopsy showed fiber size changes and centralized nuclei, suggesting a mild form of centronuclear myopathy (Koutsopoulos et al., 2013). LCCS6, found in 3 to 4 infants, showed reduced fetal movements, and antenatal ultrasound presented polyhydramnios, absent stomach, and other multiple contracture deformities. However, they did not exhibit any major renal or central nervous system malformations. The severity of polyhydramnios required patients to undergo reductive amniocentesis. The hip joints were affected and showed adducted lower limbs, symmetric deformity of the knees, flexion of hands, and foot dorsiflexion. Genetic mapping of LCCS6 patients showed that it is caused by homozygous mutation in the ZBTB42 gene (Patel et al., 2014).

Lethal congenital contracture syndrome type 7 is said to be an axoglial form of DA characterized by congenital distal joint contractures, excess of amniotic fluid, reduced fetal movements, and loss of motor function leading to death early in the prenatal period. Genetic mapping and whole-exome sequencing identified a homozygous frameshift mutation in the CNTNAP1 gene in affected individuals (Laquerriere et al., 2014). A study reported distinct clinical features associated with LCCS7, such as severe hypotonia with severe contractures and muscle wasting. Muscle biopsy of LCCS7 patients shows neurogenic muscular atrophy with reduced conduction velocities of motor nerves in the upper limbs and no responses in the lower limbs, indicating that sensory responses are absent. Brain imaging in some patients shows cerebral and cerebellar atrophy, no white matter, and smaller basal ganglia and hippocampi (Lakhani et al., 2017). LCCS8 is also an axoglial form of congenital arthrogryposis multiplex where affected patients show the presence of hypotonia, respiratory distress, facial diplegia, areflexia, and swallowing defect. Death occurs within 3 months of life owing to severe motor paralysis. It is different from LCCS7 by the absence of variability in motor nerve conduction velocity. Patients’ nerve immunohistochemistry showed Schwann cells with no myelin, and transmission electron microscopic analysis of nerve also showed no myelinated axons. LCCS8 is associated with genetic homozygous mutation in the ADCY6 gene, encoding for proteins which belong to the adenylyl cyclase family necessary for cyclic AMP production (Laquerriere et al., 2014).

Lethal congenital contracture syndrome type 9 is associated with homozygous mutation in the GPR126 gene, encoding for the G protein-coupled receptor family proteins. Clinical features show abnormal distance between the inner eye corners, upper limb DA with ulnar deviation of hands, bent fingers, sparse dermal ridges, ankylosis of the knee joint, and foot abnormality (Figure 1). Facial features include triangular face, pixie ears, depressed nasal root and bridge, thin upper lip, and micrognathia. Histologic examination of patients’ muscle biopsies shows variability in muscle-fiber diameter with small atrophic and large hypertrophic fibers. Analysis of peripheral nerves also showed an absence of myelin basic protein, indicating the defective myelination of the peripheral axons during prenatal (Ravenscroft et al., 2015). LCCS10 is caused by homozygous mutation in the NEK9 gene, encoding the never in mitosis gene a (NIM-A) family of serine/threonine protein kinases. It is associated with the presence of decreased fetal movement, multiple contractures, shortened upper and lower limbs, short broad ribs, narrow chest, incomplete lung development and protruding abdomen (Casey et al., 2016). LCCS11 is caused by mutations in the GLDN gene, a protein gliomedin necessary for the interaction between Schwann cell microvilli and axons. The pregnancies were characterized by marked polyhydramnios and fetal akinesia on prenatal ultrasound at about 27–32 weeks of gestation; however, earlier ultrasounds appeared to be normal. Clinical features also include flexion of the upper limb and extension contractures of lower limbs, as well as flexion of the wrists and fingers. Affected individuals always show pulmonary hypoplasia, sometimes retrognathia, camptodactyly, and bilateral clubfoot. Transmission electron microscopy conducted on the sciatic nerve from one of the affected fetuses demonstrated a reduced number of myelinated fibers (Maluenda et al., 2016).

## Summary

Common birth defects like congenital contractures severely complicate daily activity and cause an economic burden to the patient’s family. Arthrogryposis is one of those disorders in which patients show signs of congenital contractures in many parts of the body. DA affects the distal parts of the body, making normal life virtually impossible. Some genetic mutations reported to cause the disease were also speculated to play a role in cardiomyopathy. Genes encoding titin, for example, showed mutations responsible for developing a cardiac and skeletal disorder phenotype. Further studies are required to identify the linkage between these two disorders. Depending on the identification of suitable targets, it may be possible to treat both cardiac and skeletal muscle diseases with some form of combinatorial regimen in the future.

## Author Contributions

DD, DS, TS, and SS wrote the review manuscript. All authors approved the final version of the manuscript.

## Conflict of Interest

SS provided consulting and collaborative research studies to the Leducq Foundation (CURE-PLAN), Red Saree Inc., Greater Cincinnati Tamil Sangam, AstraZeneca, MyoKardia, Merck and Amgen, but such work is unrelated to the content of this manuscript. The remaining authors declare that the research was conducted in the absence of any commercial or financial relationships that could be construed as a potential conflict of interest.

## Abbreviations

DA: distal arthrogryposis
FSS: Freeman Sheldon syndrome
*LCCS*: lethal congenital contracture syndrome
*MYBPC1*: slow skeletal myosin binding protein-C gene
*MYBPC2*: fast skeletal myosin binding protein-C gene
*PIEZO2*: piezo-type mechanosensitive ion channel component 2 gene
*TPM2*: β-tropomyosin gene
*TTN*: titin gene.
